# How does self-compassion help people adjust to chronic skin conditions? A template analysis study

**DOI:** 10.3389/fmed.2022.974816

**Published:** 2022-10-13

**Authors:** Elaine N. Clarke, Paul Norman, Andrew R. Thompson

**Affiliations:** ^1^Centre for Behavioural Science and Applied Psychology, Sheffield Hallam University, Sheffield, United Kingdom; ^2^Department of Psychology, University of Sheffield, Sheffield, United Kingdom; ^3^School of Psychology, Cardiff University, Cardiff, United Kingdom; ^4^Cardiff and Vale University Health Board, Cardiff, United Kingdom

**Keywords:** self-compassion, template analysis, skin conditions, adjustment, qualitative

## Abstract

**Objectives:**

Skin conditions can greatly impact people's lives, but greater understanding of the processes involved in positive adjustment is required. Self-compassion has strong links to wellbeing and adaptive functioning and therefore may play an important role in adjustment to skin conditions.

**Design:**

Template analysis was used to explore how self-compassion operates in people living with skin conditions, with reference to existing theories of self-compassion.

**Methods:**

Semi-structured interviews were conducted with highly self-compassionate people with chronic skin conditions (*N* = 10). Theoretical models of self-compassion were used in the development of the initial template and interview schedule. Participants were purposively selected on the basis of having high scores on a measure of self-compassion.

**Results:**

Participants reported a variety of ongoing skin-related difficulties and their ways of managing these. Sensitivity to distress and care for wellbeing were identified as foundation themes: necessary components of a compassionate response to distress. Eleven types of difficulty-management strategies built upon these foundation themes: empathy, non-judgement, distress tolerance, self-kindness, mindful attention, perspective-taking, self-talk, self-care, using social support, concealment, and idiosyncratic coping strategies.

**Conclusions:**

Components of self-compassion helped people adjust to chronic skin conditions in a wide variety of ways, indicating that psychological adjustment is not a simple, linear process. Sometimes compassionate responses occurred automatically and sometimes with deliberate effort. Further research on compassion-based interventions for people with skin conditions is warranted.

## 1. Introduction

As well as causing physical symptoms, there is substantial evidence that living with a chronic skin condition can impact quality of life. Skin conditions have been found to affect relationships, socializing, work/school, activities of daily living, sleep, finances, and exercise (1–4). Skin conditions can also have a negative impact on mental health, including difficulties with self-esteem, body image, confidence, anxiety, and depression (1). However, there is considerable individual variation in the impact of skin conditions, for example, one study found that 35% of dermatology outpatients reported no impact of their skin condition over the previous week whereas 16% reported a very or extremely large impact (5). Individual variation in the psychological impact of skin conditions cannot be explained merely by condition severity, as previous research has found no association between clinician-assessed severity and psychological morbidity in people with acne, psoriasis and eczema (6). In contrast, self-assessed severity of the skin condition is associated with psychological morbidity (6), which highlights the important role of psychological factors in the impact of skin conditions. To reduce distress and improve quality of life, we first need to understand the psychological processes involved in living with chronic skin conditions.

Adjustment to illness has been described as “the process to maintain a positive view of the self and the world in the face of a health problem” (7, p. 1161) and emerging evidence suggests that positive psychological factors may contribute to adjustment to skin conditions. Psychological flexibility, the ability to consciously engage with the present moment in a way that is consistent with one's values, has been linked with lower appearance anxiety in people with burn injuries (8), and mindfulness and self-compassion have both been linked with lower psychological distress in dermatology patients (9, 10). Furthermore, there is preliminary evidence that mindfulness and compassion-based interventions can reduce shame and depression in people with skin conditions (11, 12). These studies indicate that positive psychological factors show promise as therapeutic targets to promote adjustment to skin conditions but further research is needed to explore how they might promote adjustment. However, previous qualitative work with people who had positively adjusted to appearance-altering conditions, which included participants with skin conditions, identified a range of adaptive strategies used, such as positive cognitive processes that de-emphasize appearance in favor of other aspects of the self (13), drawing on inner strength, using a positive outlook, and active coping (14).

The psychological concept of self-compassion as described by Neff (15), an “emotionally positive self-attitude,” may be particularly relevant to adjustment in skin conditions (15, p. 85). Self-compassion is proposed to consist of three components: self-kindness in instances of pain and failure rather than self-judgement, understanding that suffering is a shared human experience rather than feeling isolated by it; and being mindful of distressing thoughts and feelings rather than over-identifying with them (15). As self-compassion is expected to promote behaviors that enhance or maintain wellbeing (15), people with skin conditions who are high in self-compassion are expected to take appropriate steps to manage skin-related distress, thus lessening its impact. Consistent with this notion, there is evidence to suggest that self-compassion helps protect against depression in dermatological outpatients (9).

High self-compassion is expected to facilitate both problem-focused coping and emotional-approach coping (15, 16), that is, individuals with skin conditions taking practical steps to manage their physical symptoms and engaging with any emotional distress associated with the condition, respectively. In other medical populations, self-compassion has been found to be associated with active coping (a problem-focused strategy), acceptance, and positive re-framing (emotion-focused approach strategies) which were, in turn, associated with increased coping efficacy and reduced stress (17). However, living with a skin condition may present challenges that some other health conditions do not. In particular, skin conditions often cause both visible differences and physical symptoms, which can increase the complexity of living with them, and they can have a chronic intermittent course, requiring a flexible approach to their management. Therefore, it is important to research how self-compassion affects coping in a population of people with skin conditions.

Although much research on self-compassion stems from Neff's (15) conceptualization, an alternative perspective on self-compassion has been provided by Gilbert (18), based on evolutionary neuroscience. In Gilbert's model, the attributes of compassion are described as care for well-being, sensitivity to distress, sympathy, distress tolerance, empathy, and non-judgement, with the recognition that these can be directed toward the self as well as others. The conceptualizations of compassion by Gilbert (18) and Neff (15) are therefore organized around different frameworks, but do have overlapping constructs (e.g., both models include a sense of care/kindness and non-judgement). Both models have empirical support for the self-report measures (19, 20) and psychological interventions based upon them (see 21, for a review). However, to our knowledge, no previous study has explored self-compassion using components of both models.

The current study aimed to explore how trait self-compassion operates in the context of living with a chronic skin condition, using existing models of self-compassion (15, 18). Including components of both models in the current study meant that the analysis could draw on either model. The study's objective was to obtain detailed accounts of the processes involved in managing skin conditions and their impacts by conducting theoretically-informed interviews with participants with chronic skin conditions and high self-compassion.

## 2. Materials and methods

### 2.1. Participants and procedure

Participants were adult members of the general population who had chronic skin conditions and exhibited high self-compassion. The study was advertised *via* the University of Sheffield's volunteer list and social media (Facebook and Twitter). Consenting participants from a previous study (9) were also invited to take part. A purposive sample was formed by asking volunteers to complete an online screening survey, which consisted of the Self-Compassion Scale–Short Form (SCS–SF; 22), the PHQ-2 (a depression screener; 23) and questions about the skin condition. The twelve-item SCS–SF has been shown to have good internal consistency, with a Cronbach's alpha of 0.86 (22). Construct and criterion validity have been demonstrated for the two-item PHQ-2 (23). To be eligible for an interview, participants had to have an mean score of 3.75 or more on the SCS–SF and less than a total score of four on the PHQ-2. Neff (24) has stated that for self-assessment, mean self-compassion scores between 3.5 and 5 (on a 1–5 scale) can be considered “high” self-compassion. However, based on previous research (9), a more conservative cut-off value of 3.75 was used, as this represents individuals who are approximately one standard deviation above the mean for self-compassion. The PHQ-2 was used to avoid interviewing participants who were experiencing depressive symptoms, even if they were high in trait self-compassion. This was due to concerns that, as depression commonly includes negative thoughts about oneself, depressive symptoms at the time of the interview might unduly affect participants' reports of their thoughts and feelings toward themselves. On the PHQ-2, the criterion of a total score of 4 or more was considered to be the most appropriate balance of sensitivity and specificity for the current study, based on research recommendations (23). Participants also answered questions relating to the exclusion criteria: whether they had a current mental health diagnosis, a diagnosis of skin cancer, a skin condition caused by an infestation, or were seeking treatment for burns or scarring. However, it was not necessary to exclude any participants on this basis.

Eleven people participated in an interview, although one participant was subsequently excluded from the data analysis as she had not experienced her skin condition for the previous 3 years and therefore could not articulate her previous skin-related difficulties, thoughts, or feelings in depth. The other ten participants had ongoing chronic skin conditions, which required regular treatment at the time of the interviews. Participants ranged from 22 to 65 years of age. Six participants developed their skin condition in childhood, and four participants developed their skin condition as adults. Five participants had eczema, three had psoriasis, one had Darier's disease, and one had urticaria. The sample contained three men, six women, and one person who classed their gender as non-binary. Additional demographic information is shown in Table 1.

**Table 1:** Demographic and clinical characteristics of participants (*N* = 10).

**Characteristic**	***n***
Gender	
Female	6
Male	3
Non-binary	1
Employment status	
Employed/self-employed	6
Student	2
Retired	1
Unemployed	1
Relationship status	
Married	6
Non-cohabiting relationship	3
Single	1
Ethnicity	
White	9
Chinese	1
Skin condition(s)^*a*^	
Atopic eczema	4
Dyshidrotic eczema	1
Chronic plaque psoriasis	3
Guttate psoriasis	1
Darier's disease	1
Urticaria	1
Age (*M, SD*)	41.8 (13.63)
Skin condition duration, years (*M, SD*)	26.5 (14.41)
SCS–SF mean score (*M, SD*)	4.23 (0.26)
PHQ-2 total score (*M, SD*)	0.55 (1.01)

PHQ-2 = Patient Health Questionnaire 2-item depression screener; SCS–SF = Self-Compassion Scale–Short Form. Possible scores ranged from 0 to 6 on the PHQ-2 and from 1 to 5 on the SCS–SF, with higher scores indicating higher depression/self-compassion.

a One participant had chronic plaque and guttate psoriasis.

### 2.2. Data collection

An interview-specific information sheet was sent to each participant in advance and written consent to participate was collected on the day of the interview. Interviews were conducted face-to-face by the first author, who was a 33-year-old, white woman, with atopic eczema (although not generally visible to others), with clinical experience of conducting sensitive interviews. The interviewer's skin condition was not disclosed to participants to avoid unduly influencing the interviews. A reflexive journal was kept by the first author throughout data collection and analysis, which served to increase the research team's awareness of the role that might be played by pre-existing assumptions about the research topic. Interviews were semi-structured, using open-ended questions and probes as necessary. Participants were guided through describing the main impact(s) of their skin condition. Subsequent questions explored strategies that participants used to manage the difficulties of living with their skin conditions. Questions about difficulty-management strategies were structured around Neff's (15, 20) components of compassion (see Supplementary material). Interviews were audio-recorded and transcribed verbatim. Each participant was given a pseudonym to maintain their anonymity, which has been used throughout the results. The study received ethical approval from the University of Sheffield's Research Ethics Committee.

### 2.3. Data analysis

Interview transcripts were analyzed using template analysis as described by King (25), using *a priori* codes derived from the compassion literature, shown in Table 2. These consisted of theorized components of self-compassion (15, 20) and attributes of compassion (18). Template analysis was selected as the analysis method as it is a flexible approach that can incorporate both inductive and deductive (*a priori*) coding—new codes are devised and *a priori* codes are modified or deleted as fits with the data. NVivo (RRID:SCR_014802) 11 was used for coding and template construction.

**Table 2:** Compassion-related *a priori* codes used in the template analysis.

**Code**	**Description**	***n* contributing to each code**
Self-kindness^*a*^	Responding to one's suffering and personal failure with thoughts that demonstrate care, support, tenderness, patience, tolerance, and understanding toward oneself.	7
Common humanity^*a*^	Viewing suffering and personal failures as normal parts of life, and believing that feelings of failure and inadequacy are shared by most people.	7
Mindful attention^*b*^	Paying attention in a particular way (on purpose, in the present moment, and non-judgementally) either in normal daily life, during a distressing event or through meditating. Sense of watching events, thoughts or feelings pass by.	10
Care for wellbeing^*c*^	Believing that being compassionate toward oneself is a desirable attribute. Wanting to care for, nurture and support oneself to promote one's well-being.	10
Sensitivity to distress^*c*^	Being able to notice and pay attention to one's distress and needs; being attentive to changes in physical feelings, emotions and thoughts (in distressing situations).	10
Sympathy^*c*^	Being emotionally moved by one's own distress.	0
Distress tolerance^*c*^	Being able to accept and tolerate distressing feelings as they occur; being familiar with and unafraid of distressing emotions and thoughts.	9
Empathy^*c*^	Understanding one's thoughts and feelings in distressing situations.	9
Non-judgement^*c*^	Accepting and not condemning oneself for real or perceived failures or inadequacies.	10

a Concept specified by Neff (15).

b *Mindfulness* was the concept specified by Neff (15), but the label and description were modified during data analysis, as participants' statements relating to mindfulness were more reminiscent of Kabat-Zinn's conceptualization: “Mindfulness means paying attention in a particular way: on purpose, in the present moment, and non-judgementally” (41, p. 4).

c Concept specified by Gilbert (18).

An initial coding of all transcripts was carried out using the *a priori* codes. In instances where no *a priori* code was relevant, a new code was devised to encompass this data. An initial template was then produced, which was iteratively developed by comparing it to the transcripts. Codes were inserted, modified or deleted as necessary to encompass the data. In qualitative research, *saturation* is “a criterion for discontinuing data collection and/or analysis” and is commonly used to assess methodological quality (26, p. 1894). In the current study, saturation was conceptualized as an internal process, “the point at which no new information or themes are observed in the data” (27, p. 59). As such, the focus of saturation was on the data analysis rather than data collection. Development of the template ended when saturation of data analysis was considered to have been achieved: when all data were codable using the template (25) and no new codes were emerging from the data (27). The final template was used to interpret findings from the data. An audit trail was kept of the developing templates, showing how the final interpretation of the data was produced (28). This included the use of a codebook of all codes that were applied to the data, which documented when codes were inserted, modified, merged or deleted. To demonstrate methodological rigor, an audit of the data analysis was conducted by the third author, which included cross-checks between identified themes and interview transcripts (29).

## 3. Results

Participants reported a wide range of difficulties associated with their skin conditions, consisting of physical symptoms and psychological, social, and practical impacts. All participants experienced negative thoughts and/or emotions relating to their skin condition and all talked about the reactions of others (e.g., strangers or acquaintances) to seeing their skin condition. Differences between past and present impacts were common, with participants' skin conditions often having had more negative impact in the past.

All of the strategies that participants used to manage the difficulties of living with a skin condition were built upon two basic components: sensitivity to distress and care for wellbeing. As such, these were described as *foundation* themes, in that they were the necessary components of a compassionate response to distress. Eleven difficulty-management strategies that built upon sensitivity to distress and care for wellbeing were identified: non-judgement, mindful attention, perspective-taking, empathy, distress tolerance, self-kindness, self-talk, self-care, using social support, concealment, and idiosyncratic coping strategies. These strategies contained cognitive and behavioral elements: participants' attitudes toward themselves, their condition, and the wider world, and choices that required ongoing, deliberate action.

### 3.1. Foundation themes

#### 3.1.1. Sensitivity to distress

Participants were generally very good at being sensitive to their own distress, whether this was physical symptoms or emotional distress. Once participants had noticed their distress, they then applied one or more specific strategies to try to alleviate it. They were often able to see their distress as a cue for taking holistic remedial action, that is, not simply attending to the skin but to their lives more generally.

I just see it [psoriasis] as my body telling me that things aren't right, my systems aren't coping, so when it happens I try to think ‘right, what can I do to bring my body back into alignment?' (Joanne)

Participants were also able to use sensitivity to distress as a preventative measure: being sensitive to distress had allowed participants to become aware of helpful and hindering factors for their skin conditions, and so could make appropriate choices to try to prevent flare-ups. Furthermore, having sensitivity to distress contributed to participants being skilled at articulating their distressing thoughts and feelings, although often these were historic.

I think at that time …it [urticaria] did get me really down, ‘cos I thought ‘I've got no control over it, you know, I'm gonna lose my job because I can't work….' (Julie)

#### 3.1.2. Care for wellbeing

Behind all the strategies that participants were using to manage their difficulties was a sense of valuing themselves and a consistent desire to look after themselves well.

[How I treat myself is] just giving myself a bit of space and being kind to myself a bit, doing things that I know probably make me feel good, like go for a run. Yeah.…if I need a bit of peace then I let myself have some peace. So being kind to myself I think. (Helen)

At times, this care for wellbeing required finding a balance between actions that would benefit their physical health and those that would benefit their mental/emotional health. Sometimes this balance also involved choices between short-term and long-term wellbeing.

### 3.2. Difficulty-management strategies

#### 3.2.1. Non-judgement

All participants expressed and/or demonstrated non-judgement about the skin-related difficulties they experienced. They were often able to talk about their perceived failures and inadequacies without condemnation.

I guess it [eczema] makes me feel kind of …like I'm missing out. But I don't feel like (pause) I'm of less self-worth. (David)

As part of this non-judgement, participants very commonly expressed an acceptance of their condition, appreciating the futility of wishing that things would be or could have been different.

I wouldn't change it for instance.…I've just sort of accepted who I am. …That isn't to say that I don't want it to go away or get better, I do want it to get better but I wouldn't change my life history or who I was ‘cos that's part of me now. (Martin)

Participants also often reported a lack of self-consciousness about their condition, which in many cases had developed over time. The appearance of the skin was generally less salient for participants than when the condition first developed.

#### 3.2.2. Mindful attention

All participants used some form of mindfully paying attention to the present moment and were aware that this helped them deal with potentially distressing situations. Sometimes this mindful attention was achieved through formal meditation practices, such as observing the breath or letting go of thoughts and feelings without reacting to them. At other times, mindful attention was used more informally, through focussing attention on current activities, that is, acting with awareness.

But, you know, just concentrate on actually what you're doing at that time and things like…if you're washing pots or anything, just feeling how water feels on your skin…and I think that is, that has been a massive help as well. (Julie)

Some participants noted that they found it easiest to use mindful attention while doing yoga or sensory activities, and therefore made time for these. Not paying undue attention to the skin condition and generally having a present-moment focus was also helpful for some.

I don't really think about it [psoriasis] too much to be honest. (Steve)

#### 3.2.3. Perspective-taking

All participants were able to reflect on their skin-related difficulties from other perspectives, particularly those relating to other people and other times in their lives, and they did this with apparent ease. As a result of these perspective-taking skills, all participants spontaneously expressed a sense of fortune or gratitude for the good things in their lives; very commonly this was for having a supportive family and that the skin condition was not worse in some way. Sometimes this sense of fortune/gratitude included the use of downward social comparison; the appreciation that things are worse for some other people.

…[I]t kind of eases my stress a bit, I guess, to know that people have it worse than me and they're still, they're still living, right. (David)

Common humanity, the understanding that suffering is a normal part of life, was also demonstrated by many participants.

I mean everybody's got their own problems haven't they, just because you haven't got a skin condition it doesn't mean that you haven't got your own set of problems. (Philippa)

Being able to see their skin-related difficulties from different perspectives meant that participants saw their skin condition as just one aspect of their lives, even though it was difficult to live with at times.

#### 3.2.4. Empathy

Participants were able to understand thoughts and feelings that occur in distressing situations. Participants most often explicitly expressed empathy in the context of understanding others' difficulties, or potential difficulties.

I think if you were someone that was quite concerned with how you look and that kind of thing I could see that [psoriasis] would affect you a lot more because it'd be (pause) you'd be more conscious and more worried about it I think. (Steve)

However, participants also had good understanding of their own thoughts and feelings about skin-related difficulties. They could use this understanding to decide the best way forward for them, even if it contravened medical advice at times. Having empathy for one's own difficulties—both physical and emotional—contributed to finding the right balance between short-term and long-term consequences of lifestyle choices.

#### 3.2.5. Distress tolerance

Participants very commonly showed distress tolerance: they were able to accept and tolerate distressing feelings and could therefore actively engage with distressing situations, or potentially distressing situations, rather than try to escape or avoid them. For example, some participants chose to exercise despite pain/discomfort due to sweat aggravating the skin condition, and some chose to go into social situations despite feeling self-conscious around others.

This ability to tolerate distress fed into participants' abilities to get on with their lives despite their skin conditions: they frequently chose to do valued activities even though this meant having to accept negative consequences due to the skin condition.

But I would never say [that] eczema would be a reason why I wouldn't go somewhere or do something. …like I might, you know, mentally make a few calculations about the pros and the cons but generally there are much *bigger* pros than cons. (Helen)

Another aspect of distress tolerance was willingness to allow limited periods of time to be upset about skin-related difficulties. After this, participants felt able to get back on with their lives.

If [my skin]'s really, really, really bad, I'll just shut [myself] off …say half an hour, and then I think ‘come on, buck up' …[and then] I'm alright. (Maureen)

#### 3.2.6. Self-kindness

Participants often reported responding to their difficulties with thoughts that showed self-kindness, that is, directing care and support toward themselves. This self-kindness occurred naturally, without too much conscious effort. However, some participants had experienced previous difficulties with mental health problems and/or self-criticism, which they had worked through to arrive at their current attitude of self-kindness. For other participants, self-kindness seemed to have developed naturally earlier in life.

That counselling…gave me some really good kind of basic tools around not beating yourself up, …treat yourself how you'd treat other people. (Emily)

#### 3.2.7. Self-talk

Participants very commonly ‘had a word with themselves' when experiencing skin-related problems. When doing this, they were deliberately directing their thoughts in helpful ways and reminding themselves of their coping strategies, often involving trying to take a different perspective.

I do often think to myself, you know, ‘Will this matter in 5 years' time? Will this be important?' (Claire)

As part of their self-talk, participants commonly incorporated a problem-solving approach/wisdom: considering options and being prepared to find out what is helpful.

[I think about] how to manage. …So that could be like medical interventions, what exactly am I going to do medically or biologically to help myself, so am I drinking enough, am I eating the right foods, am I getting enough sleep, anything that will affect my body chemistry. So I think; I strategise. (Martin)

This approach helped participants address the tensions between different choices they might make in terms of their physical and emotional health. It also helped participants respond flexibly to situations, meaning that they could take their current circumstances into account rather than always responding to situations in the same way.

#### 3.2.8. Self-care

All participants used a variety of self-care strategies, in which the sole aim was to look after oneself. Self-care activities either focused on looking after physical health, particularly the skin condition, or were more holistic, leisure activities that incorporated care for emotional health as well, as described below.

##### 3.2.8.1. Physical health care

All participants talked about the specific strategies they used to manage the physical symptoms of their skin conditions. These strategies fell into two categories. First, all participants took steps to manage their skin conditions on a daily basis. Most often this was through the use of moisturizers.

[Moisturising is] such an everyday part of my life I don't really see it as management, I just see it as part of my everyday life. (Helen)

Using specialist cleansing products, avoiding scratching, taking immunosuppressant medication, and following special diets were other daily management strategies used by participants.

Second, most participants made sure they addressed flares promptly, through a variety of means: increased use of moisturizers or steroid creams, taking antihistamines or steroid tablets, using phototherapy (UVB or PUVA treatments), and by cooling the skin.

All participants chose to avoid certain things that triggered or exacerbated their skin conditions, but these were usually only things that were not highly valued. Most commonly this was avoiding certain physical activities.

I try and avoid [swimming] just ‘cos I just don't like the feeling of it on my skin. …but because I don't really like swimming anyway, it doesn't really bother me. (Helen)

Occasionally, participants chose to miss out on valued activities, for example, going away with friends, because of their skin condition and this had a more negative impact on them. However, avoiding such activities was unusual, indicating that participants were responding flexibly to fluctuations in their health.

Sometimes participants avoided social situations because they felt too ill/tired due to their skin condition and wished to protect their health from the demands of socializing. Participants commonly tried to avoid known environmental triggers such as sunlight, heat, pollution, dust, and hard water. Certain fabrics, cosmetics, jewelery, foods, and drinks were also avoided. Although avoiding physical triggers helped minimize the severity of the skin conditions, these choices were not without negative consequences, making normal activities feel difficult.

I personally can't put much makeup on anymore…so it's quite hard to get excited about going out and socialising when you're not quite as dressed up as everybody else. (Philippa)

Some participants also tried to make sure that they ate healthily (e.g., eating plenty of fruit and vegetables) with the aim of improving their general health, and therefore their skin condition.

##### 3.2.8.2. Leisure activities

Most participants deliberately made time to do enjoyable activities to look after themselves emotionally. Some of these activities were relaxing: reading, knitting, puzzles, baths, reflexology; while others were more active: cooking, playing games, trips out.

[To manage how I'm feeling] I'll make time for myself. I'll read, 'cos I love reading, and I think when you, when you have got other things on your mind, …you tend not to make time for yourself and I think it's important to just make that time, so I started doing a bit of knitting…. (Julie)

Sometimes there was overlap between the activities that people did anyway and those that they did as a way of improving/maintaining their mood. In these cases, participants made sure that they carried on doing their enjoyable activities despite their skin conditions.

Participants commonly allowed themselves to rest when their skin condition flared, although work commitments could make this feel difficult. Sometimes participants rearranged their work or study schedules to facilitate extra rest.

[O]kay can I do some, you know, later starts or early finishes [at work] to give myself a bit more time…(Emily)

Some participants with skin conditions that were exacerbated by stress used exercise as a way of caring for themselves. Exercise helped to relieve stress and therefore this had a positive effect on both their mental and physical health.

#### 3.2.9. Using social support

Participants commonly used one or more types of social support to help manage skin-related difficulties. All participants had people in their lives who they described as supportive. Most commonly, participants found it helpful to talk to their significant others about skin-related difficulties, although this was needed infrequently as participants' skin conditions were generally under good control. Some participants identified that simply spending time with others helped them to feel better when they were experiencing skin-related difficulties. When they did this, the focus was not on the skin condition but other everyday things.

[S]ometimes it's nice having somebody there just to sit and like watch the telly with, or sit and chat to, or go out for a walk with or something like that. (Philippa)

Some participants had used online support, as this was a convenient way of connecting with others with the same skin condition who therefore understood the difficulties involved. Some participants now used online forums to provide support to others who were going through similar difficulties.

#### 3.2.10. Concealment

Although participants were generally coping well with their skin conditions, concealment was commonly used as a strategy for managing potential social difficulties. However, concealment was viewed as a choice, with participants stating that if they did not want to cover their affected skin, they would not. Concealment was only used when convenient, but having the option to conceal seemed to lessen the impact of the skin condition.

Because I can just wear a shirt and trousers like this at work and no-one asks me about it I kind of can just get on with it. (Steve)

Some participants gave examples of going to greater lengths to conceal their skin condition in the past. Often, this consisted of wearing clothes contrary to their normal preferences in hot weather or on special occasions. However, better control of the skin condition and a change in mindset over time meant that participants no longer felt the need to conceal the skin condition to this extent.

#### 3.2.11. Idiosyncratic coping strategies

Four participants reported idiosyncratic strategies to reduce distress during skin flares. These consisted of using an autonomous sensory meridian response (ASMR), cleaning and tidying, and reading scientific research. All of these strategies were underpinned by being sensitive to distress and caring for wellbeing, but were used by so few participants they could not be incorporated into other themes.

## 4. Discussion

This study sought to investigate how self-compassion may operate in adjustment to chronic skin conditions. Participants were highly motivated to take good care of themselves, both physically and psychologically, and were proactive in taking measures to promote and maintain their wellbeing. However, participants still experienced difficulties and psychological distress in connection with their skin conditions, including negative automatic thoughts about their skin from time to time. However, none of them *tried* to think this way, or believed their overly negative thoughts to be accurate on reflection: when they spoke of subsequently ‘talking to themselves' it was with helpful, compassionate thoughts. Compassionate thoughts about the skin condition can therefore occur in (at least) two contexts: as compassionate automatic thoughts and as deliberate compassionate ‘self-talk' after noticing distress. Some participants had arrived at their current level of self-compassion after overcoming difficulties with anxiety, depression or habitual self-criticism, indicating that such difficulties can provide opportunities for personal growth.

The highly self-compassionate participants in the current study reported using a variety of strategies to deal with the difficulties of living with a skin condition: non-judgement, mindful attention, perspective-taking, empathy, distress tolerance, self-kindness, self-talk, self-care, using social support, and concealment. These strategies may, therefore, constitute adaptive responses for living with skin conditions (although it should be noted that concealment, which can be maladaptive (30), was used in a specific way by participants in this study: as an active choice when it was convenient). Furthermore, all of the difficulty-management strategies were built upon sensitivity to distress and care for wellbeing, either explicitly or implicitly (e.g., if a participant was choosing to rest, this implies an awareness of one's physical needs and the desire to look after oneself). The importance of sensitivity to distress and care for wellbeing suggests that these abilities may be particularly adaptive for people with skin conditions.

More broadly, the current findings suggest that self-compassionate responses to life difficulties are complex and people can and do vary in the compassionate attributes in which they have strengths. For example, some participants appeared to be highly distress tolerant whereas others had strong perspective-taking skills. This suggests potential for compassion-based interventions to help people build on strengths they already possess and develop new skills that are lacking. Indeed, existing compassion-based interventions typically incorporate a variety of techniques (e.g., see 31–33) and the current findings support the benefit of this.

The current findings also offer insights about the relative importance of the different concepts within models of self-compassion. Within Gilbert's (18) conceptualization, sensitivity to distress and care for wellbeing emerged as vital ingredients for self-compassion. Three other components, distress tolerance, non-judgement, and empathy, also played important roles in compassionate responses to skin conditions. However, the final attribute of compassion in this model, sympathy, did not contribute to difficulty-management strategies. Participants never talked about feelings of sympathy for their own distress; rather, their focus was on managing the problem or looking after themselves. It is possible that being sympathetic to one's own distress is implied through care for wellbeing, but sympathy did not translate to any practical strategies for these participants. This finding is consistent with research exploring compassionate attributes expressed by people undertaking a compassion intervention (34). In Gilbert's model (18), sympathy is defined as being emotionally moved by distress, while empathy consists of understanding distress (i.e., understanding the thoughts and feelings connected to it, and why these have arisen). However, Sommers-Spijkerman et al. (34) found that sympathy tends to be expressed alongside empathy rather than on its own, leading the authors to propose a simplified model that incorporates sympathy in the concept of empathy.

Within Neff's conceptualization of self-compassion (15), self-kindness and mindfulness emerged as having greater importance than common humanity. While participants often spontaneously talked about concepts relating to mindfulness and self-kindness, common humanity was less salient, and emerged as part of a higher-order theme of perspective-taking. Participants' use of online peer support could be argued to be partly the result of having a sense of common humanity, that is, knowing other people experience similar difficulties and seeking them out for support. However, due to the small sample and qualitative nature of the study, these findings remain tentative. Further research is needed replicate and quantify these findings, and to explore whether the importance of the components of self-compassion in each of the models varies in different populations.

### 4.1. Strengths and limitations

A key strength of the study was identifying participants who had lived with their skin condition for a number of years, which meant that they had had time to adjust and so refine their difficulty-management strategies. The use of template analysis was a further strength, as this meant that themes that emerged during analysis could include, but were not limited to, existing concepts from the compassion literature.

A potential limitation was that two of the authors have personal experience of eczema and pre-existing interest in the role of positive psychological variables, which may have influenced data collection and analysis due pre-existing ideas. However, the impact of any preconceptions was mitigated using various quality control processes, including use of a semi-structured interview schedule, use of a reflexive diary, team debriefing within supervision, and use of an audit trail of the codebook and iterative templates. These processes helped to guard against the research team's experience and expertise in the topic exerting an undue influence on the findings (29).

A limitation of this study was that participants had a relatively small range of skin conditions. A notable absence was acne, which, along with eczema and psoriasis, is one of the most common conditions seen by dermatologists in the UK (35). As psychological experiences may vary with skin condition, the findings of the current study require replication with people who have acne and in a larger sample, given the small sample size.

### 4.2. Implications

The findings of the current study indicate that self-compassion plays a role in adjustment to chronic skin conditions and is therefore an appropriate therapeutic target for alleviating psychological distress in this population. Findings further suggest that interventions to increase self-compassionate responding in people living with skin conditions should have two key targets: (1) increasing sensitivity to the distress that results from having the skin condition, so that remedial action can be taken, and (2) developing effective care for wellbeing that can negotiate between emotional and physical health demands, and between short-term and long-term wellbeing. Several different compassion-based interventions exist (see 21, for a review) and have been shown to be effective for treating psychological distress (36, 37), including in people with chronic physical health conditions (38). Furthermore, self-compassion interventions have been found to improve the self-regulation of health behaviors (39) and there is emerging evidence that compassion-based interventions can benefit people with skin conditions (11, 12, 40). The current findings provide an increased understanding of how self-compassion translates into adaptive strategies for managing the challenges of living with a skin condition.

### 4.3. Conclusions

In the context of living with a chronic skin condition, the key processes involved in self-compassion were having sensitivity to skin-related distress and caring for one's physical and mental wellbeing, with a variety of other adaptive strategies being built upon these: non-judgement, perspective-taking and empathy with respect to skin-related difficulties; mindfully attending to the present moment; tolerance of skin-related distress; kind automatic thoughts and deliberately helpful self-talk in response to skin-related difficulties; physical and emotional self-care activities; spending time with others who are supportive about the skin condition; concealment of the skin as a choice; and using idiosyncratic coping strategies.

## Data availability statement

The original contributions presented in the study are included in the article/Supplementary materials, further inquiries can be directed to the corresponding author/s.

## Ethics statement

This study involving human participants was reviewed and approved by the University of Sheffield Department of Psychology Research Ethics Committee. The participants provided their written informed consent to participate in this study.

## Author contributions

EC, PN, and AT contributed to conception and design of the study. EC collected the data and performed the analysis and wrote the first draft of the manuscript. AT conducted the audit of the data analysis. All authors contributed to manuscript revision, read, and approved the submitted version.

## Funding

This research was supported by a Faculty of Science Scholarship at the University of Sheffield.

## Conflict of interest

Author AT is a topic editor of the Frontiers Research Topic ‘Psychosocial Aspects of Skin Conditions and Diseases', was the lead psychological advisor to the recent mental health report produced by the APPGS and has also received honorariums and/or research support from pharmaceutical companies involved in the treatment of skin conditions (including UCB, Novartisis, and Pzifer). The remaining authors declare that the research was conducted in the absence of any commercial or financial relationships that could be construed as a potential conflict of interest.

## Publisher's note

All claims expressed in this article are solely those of the authors and do not necessarily represent those of their affiliated organizations, or those of the publisher, the editors and the reviewers. Any product that may be evaluated in this article, or claim that may be made by its manufacturer, is not guaranteed or endorsed by the publisher.
