# Supplementary material for: How does self-compassion help people adjust to chronic skin conditions? A template analysis study
Source: Front Med (Lausanne). 2022 Oct 13;9:974816. doi: 10.3389/fmed.2022.974816 (PMC9606614; doi:10.3389/fmed.2022.974816)
Supplement: Supplementary file 1 [file Data_Sheet_1.pdf]

Interview Schedule

Exploring the effects and impact of skin condition (to provide context for later questions).	**Can you tell me about the last time your [skin condition] was particularly bad?**	• When was it?<br>• What happened?<br>• What was your skin like at that time?
	**How did it affect your life at the time?**	• Did it stop you from doing anything? (e.g. work/ leisure/home/socialising/relationships)<br>• Did it make it difficult to do your normal activities?<br>• *If multiple impacts:* Which one of those things bothers you most?
	**Have there been times when your [skin condition] affected you in any other ways**?	• *If yes:* How did it affect you? (*explore as above)*
Participants' attitudes and responses towards skin-related difficulties: exploring self-kindness vs self-judgement	*For main areas of difficulty reported:*<br><br>**When things are difficult because of your [skin condition]** *(refer to experiences reported)***, how do you feel?**	• People usually experience some negative emotions when they have difficulties in life. What negative emotions come up for you?
	**When [X occurs] because of your [skin condition], what goes through your mind?**	• How do you think about the difficulties?<br>• What do you tend to think about when things are tough with your skin?<br>• How do you make sense of what's happening?
	**What are you like with yourself when things are difficult because of your [skin condition]?**	• Does [impact] affect the way you think about yourself? If so, how?<br>• How do you treat yourself when your [skin condition] is causing problems for you?<br>• When things are difficult/when you're feeling X because of your [skin condition], what do you say to yourself about yourself?<br>• If you were to pause and reflect on how you feel when your [skin condition] is worse, how do you think you'd feel about yourself?
	**When [X occurs] because of your [skin condition]** *(refer to experiences reported)***, what do you actually do?**	• How do you try to manage those problems?<br>• When you have X problem, what happens after that?<br>• How do you look after yourself when your [skin condition] is bad?
	**How does that affect you?**	• Does doing X help? If so, how?<br>• What happens after you do that?<br>• How does that style of treating yourself affect the way you feel?

Exploring mindfulness vs over-identification	**When you feel [X] because of your skin condition, how do you respond to those feelings?**	• What happens when you start to feel like that?<br>• What do you do when you feel X?<br>• How much time do you spend thinking about the problems due to [skin condition]?
	**How does that affect your feelings?**	• What happens after that?<br>• Does doing that make you feel better, worse, or no different?<br>• Does it have the same effect in the short term and the long term?
Exploring common humanity vs isolation	**How do you think the problems you've had due to [skin condition] compare with other people's life experiences?**	• When things are difficult because of your [skin condition], how do you think that compares to other people's difficulties?<br>• Do you think that your problems are similar to or different from most other people's?<br>• How do you think your experiences compare to most people's?<br>• How do your experiences compare to those of other people who have [skin condition]?
Exploring development of approach to managing skin condition	**How do you think you developed that way of managing the feelings of [X] that come with your [skin condition]?**	• Where has that way of managing those feelings come from?<br>• What experiences have contributed to your current way of dealing with things?<br>• What did you learn from that?<br>• Has the way you manage the problems that come with your skin condition changed over time? *If yes:* What do you think has led to that?
	**Do you use the same sort of approach to cope with other problems in your life?**	• Where has that way of managing those feelings come from?<br>• What experiences have contributed to your current way of dealing with things?<br>• What did you learn from that?<br>• Has the way you manage the problems that come with your skin condition changed over time? *If yes:* What do you think has led to that?
	**Is there anything that you would still like to change about the way you manage your skin-related problems?**<br><br>*If yes:* **How do you think that would affect you?**	• How do you tend to approach problems in other areas of your life?<br>• Has having your [skin condition] affected the way you approach other sorts of problems?

Exploring interpersonal flows of compassion: context for what strategies participants value	**How do you tend to respond when someone close to you is struggling with a problem in their life?**	• Are there any problems caused by your [skin condition] that you are still struggling to manage?<br>• What would you like to do differently?
	**How does that compare to the way you treat yourself?**	• What do you do to help them?
	**What have other people said or done when you've been struggling with your skin that you've found helpful?**	• When it's you that is having a problem, rather than someone else, do you respond in the same way?
Other	**Are there any other comments you'd like to make about your skin condition or how you manage it?**<br>**Is there anything important we haven't covered?**	• What was helpful about that?<br>• How did that affect you?

Notes:

Participants' own words were substituted for [skin condition], [X] and [impact] during the interviews.

Questions in **bold type** were asked of each participant, in a manner that fit the flow of conversation and incorporated their previous answers.

Bullet pointed questions were asked as optional additional questions if participants did not understand the initial question and needed it to be re-phrased.

Instructions for the interviewer relating to the flow of the interview are in *italicised type*.

Depending on the participant's experiences, difficulties that coming with the skin condition might be physical symptoms (e.g. itch), impact on activities of daily living (e.g. avoidance of activities that exacerbate symptoms) or psychological difficulties (e.g. feeling self-conscious because of the skin condition). The interviewer clarified the focus of questions as needed throughout the interview.

General prompts were used as necessary to help the participants talk about their experiences, such as:

- Can you tell me more about that?
- What was that like?
- What happened next?

Reflection and clarification of what the participants had said was used throughout the interviews.
